# Dose-Dense Epirubicin and Cyclophosphamide Followed by Weekly Paclitaxel in Node-Positive Breast Cancer

**DOI:** 10.1155/2014/259312

**Published:** 2014-09-09

**Authors:** Hamid Reza Mirzaei, Fatemeh Nasrollahi, Ladan Mohammadi Yeganeh, Sepideh Jafari Naeini, Pegah Bikdeli, Parastoo Hajian

**Affiliations:** Department of Radiation & Oncology, Cancer Research Center, Shohadaye-Tajrish Hospital, Shahid Beheshti University of Medical Sciences and Health Services, Tehran, Iran

## Abstract

*Background*. Adding taxanes to anthracycline-based adjuvant chemotherapy has shown significant improvement in node-positive breast cancer patients but the optimal dose schedule has still remained undetermined. *Objectives*. The feasibility of dose-dense epirubicin in combination with cyclophosphamide (EC) followed by weekly paclitaxel as adjuvant chemotherapy in node-positive breast cancer patients was investigated. *Methods*. All patients were treated with epirubicin (100 mg/m^2^) and cyclophosphamide (600 mg/m^2^) every two weeks for four cycles with daily Pegfilgrastim (G-CSF) that was administered 3–10 days after each cycle of epirubicin and cyclophosphamide infusion which followed by (80 mg/m^2^) paclitaxel for twelve consecutive weeks. *Results*. Sixty consecutive patients were analyzed, of whom 57 patients (95%) completed the regimen and no case of toxicity-related death was observed. Grade 3/4 hematologic toxicity was uncommon and the most common grade 3/4 nonhematological adverse event was neuropathy disorders. *Conclusions*. Dose-dense epirubicin and cyclophosphamide followed by weekly paclitaxel with G-CSF support is a well-tolerated and feasible regimen in node-positive breast cancer patients without serious complications.

## 1. Introduction

Anthracyclines are the most effective drugs in the treatment of breast cancer, and the addition of a taxane to an anthracycline-containing regimen, after or concurrently with anthracycline treatment, appears to provide significant benefit, particularly in node-positive cases [1–3] and the combination of paclitaxel with anthracycline has been reported as an active regimen in improvement of disease-free survival and overall survival [4, 5]. A large number of adjuvant taxane studies have been reported. The CALGB 9141 and NSABP B-28 trials demonstrated that doxorubicin and cyclophosphamide (AC) plus paclitaxel (PAC) is superior to four cycles of AC alone, which in turn has equivalent efficacy to six cycles of cyclophosphamide plus methotrexate and 5-fluorouracil (CMF) [6, 7].

A randomized multicenter phase III study was conducted by Polyzos et al. to compare the sequential docetaxel followed by epirubicin/cyclophosphamide combination with that of epirubicin, cyclophosphamide, and 5-fluorouracil (FEC). The sequential docetaxel followed by epirubicin/cyclophosphamide adjuvant chemotherapy regimen resulted in improved five-year disease-free survival (DFS) in women with axillary node-positive early breast cancer [8].

Also, higher doses (dose intense) and more frequent administration of these drugs (dose-dense) were well tolerated and correlated to disease-free survival in breast cancer [9–11]. One method for increasing dose intensity in high risk patients in order to achieve the most benefit of maximum dose intensity is reducing the conventional drug dose intervals (dose-dense regimen).

Clinical trials suggested that paclitaxel was more effective and less myeoltoxic taxane than docetaxel and the low-dose weekly paclitaxel might be superior to higher doses given less frequently in both metastatic and adjuvant setting [4, 5].

The cancer and leukemia Group B trial 9741 compared different sequential schedules of doxorubicin, cyclophosphamide, and paclitaxel given either every 3 weeks (conventional) or every 2 weeks (dose-dense) with systemic granulocyte colony-stimulating factor (G-CSF) support in the dose-dense arms. The dose-dense regimens significantly prolonged both disease-free survival and overall survival without increasing toxicity [11].

In a published trial, women with early breast cancer received four cycles of doxorubicin and cyclophosphamide every 3 weeks postoperatively and then they were allocated to weekly taxane versus 3-weekly taxane and paclitaxel versus docetaxel in a factorial design. They concluded that treatment with doxorubicin and cyclophosphamide followed by weekly paclitaxel is associated with improved disease-free survival and overall survival in comparison with paclitaxel given every 3 weeks [5].

Previous clinical trials and meta-analysis showed that epirubicin is effective as doxorubicin but with less cardiotoxic and myelosuppressive effects [12, 13].

Since the optimal schedule of administration of epirubicin and cyclophosphamide plus paclitaxel and sequence-dependent toxicity have not been elucidated yet, further clinical trials with higher sample size are suggested.

We conducted this trial to evaluate the safety and feasibility of dose-dense epirubicin and cyclophosphamide followed by weekly paclitaxel as adjuvant chemotherapy in node-positive breast cancer patients.

## 2. Patients and Methods

### 2.1. Patients' Eligibility

Eligible women were between 18 and 70 years of ages and had undergone primary surgery (i.e., mastectomy or lumpectomy), with histologically proven invasive breast cancer and at least one histologically resected positive auxiliary lymph node. Eastern Cooperative Oncology Group performance 0-1, adequate biological functions (hemoglobin > 10 g/dL; absolute neutrophil count > 1.5 × 10^9^/l; platelets > 100 × 10^9^/l; serum creatinine clearance > 60 mL/min; bilirubin < upper normal limit (UNL); alkaline phosphatase (ALP) < 5 × UNL; and aminotransferases < 2.5 × UNL), and normal cardiac function were confirmed by left ventricular ejection fraction (LVEF > 50%).

Patients were excluded if they had even one of the following: T4 stage, inflammatory breast cancer, ductal carcinoma in situ (DCIS), prior history of any other cancer or anticancer therapy, other significant medical conditions (most notably cardiac, neurologic disorders), sensory or motor neuropathy of severity greater than WHO grade 1, pregnant or breast-feeding patient or inadequate contraception, or any other condition that was considered to make the patient ineligible for this study by the investigators. The study was performed in accordance with the declaration of Helsinki and written informed consent was obtained prior to participation in the study. The study protocol was reviewed and approved by institutional review board of the Shohadaye-Tajrish Hospital.

### 2.2. Patient Assessment

Eligible patients who had given consent were invited to attend the assessment to provide baseline data as follows: full medical history and physical examination, hematology and biochemistry assessment (such as renal and liver function tests), hormone receptor status, chest radiography and/or computed tomography (CT) scan, electrocardiogram and echocardiography, abdominal and pelvic ultrasound or computed tomography (CT) scan, bone scan, and other evaluation based on symptoms of patients.

### 2.3. Treatment Plan

All patients received epirubicin (100 mg per square meter of body-surface area, given by slow intravenous push during a period from 5 to 15 minutes) and cyclophosphamide (600 mg per square meter in 300–400 cc normal saline solution by intravenous infusion from 30 to 60 minutes) every two weeks for four cycles. Followed by weekly paclitaxel was given as a 1-hour intravenous infusion via 300 cc normal saline solution at a fixed dose of 80 mg of per square meter for twelve cycles. Granulocyte colony-stimulating factor (G-CSF) 300 microgram daily was administered in all patients on days 3–10 of each course of epirubicin and cyclophosphamide.

Premedication for EC consisted of a 5-HT3 serotonin receptor antagonist (e.g., granisetron or ondansetron) and dexamethasone intravenously. Standard premedication with glucocorticoids, H1 and H2 receptor blockers (e.g., promethazine, clemastine, and ranitidine) were given before paclitaxel administration. Actual body weight was used for body-surface area calculations. A complete blood count with leukocyte differential was performed before each chemotherapy treatment. Patients were seen every week during treatment for history and physical examination and assessment of performance status and toxicity.

### 2.4. Dose Modification

Treatment was given on day 1 of every cycle if absolute neutrophils count (ANS) and platelets were ≥1.5 × 10^9^/l and ≥100 × 10^9^/l, respectively. Unless doses in the subsequent cycle were reduced, doses in the current cycle were administered according to protocol. In case of grade 4 nonhematologic toxicities (excluding nausea, vomiting, and alopecia), treatment was delayed by up to one week, and complete blood count and toxicity grading were repeated weekly. Patients requiring a treatment delay of more than three weeks were removed from the study or treatment continued after patients' adequate recovery.

After chemotherapy completed, radiation therapy was conducted following the last cycle of chemotherapy and after recovery from any toxicity, according to standard institutional dosing guidelines and techniques. Patients whose tumors expressed either (or both) the estrogen or progesterone receptor-positive were placed on a 5-year course of tamoxifen 20 mg/day. Postmenopausal patients were offered aromatase inhibitors as an alternative to tamoxifen.

### 2.5. Evaluation of Toxicity

Toxicity for each cycle was assessed before the commencement of the following cycle and was graded using the National Cancer Institute Common Toxicity Criteria (NCI-CTC version 3).

Patients were followed by history and physical examination within 15 days and one month after last infusion, and this follow-up was processed every 2-month intervals for the first year of chemotherapy completion then every six-month interval for years 4-5. Each visit included a complete blood count, along with hematologic studies and chemistries (liver and renal function tests), chest X-ray, and ECG. Computed tomography scan of the chest, abdomen, and pelvic and a bone scan (or both) were considered if clinically indicated abnormal laboratory values at the discretion of the physician. After treatment completed, echocardiography was carried out in all patients. Mammography was performed on the remaining breast(s) annually.

### 2.6. Statistical Methods

The objective of the study was to evaluate the toxicity of EC plus paclitaxel in node-positive breast cancer and to answer the trial aims, 60 eligible patients were required. Descriptive methods were applied for all the variables. Statistical analyses were carried out using SPSS software version 16 and *P* < 0.05 was considered significant.

The primary endpoint was the incidence (*r*) of grade 4 toxicity. The study was designed as a one-stage three-outcome phase II study, in which H0 was *r* > 50% and HA was *r* < 25%. Under these assumptions and with *α* and *β* errors rate of 5% each, 60 patients were assigned to reject a toxic treatment (with >50% grade 4) and accept a nontoxic treatment (with <25% grade 4) with a probability >90%. If <15 grade 4 toxic events occurred, the treatment was to be considered tolerable. If >30 grade 4 toxic events occurred, the treatment was to be considered intolerable. If 16–29 grade 4 toxic events occurred, the study was not conclusive.

## 3. Results

### 3.1. Patient Characteristics

Sixty eligible patients were enrolled into the study from April 2007 to March 2009 in Shohadaye-Tajrish Hospital. Patient characteristics at the time of entering the study are listed in Table 1. The mean age of the patients was 49.6 years and 58.3% of the patients were <50 years old. The median number of examined lymph nodes was 11 (range 2–37). 58.6% of the patients had one to three positive lymph nodes, 27.8% had four to nine positive nodes, and 13.6% had ten or more positive nodes. Median tumor size was 3 cm and in 28.3% of the patients, size of tumor was less than ≤2 cm in maximum diameter. The tumor was positive for both of estrogen and progesterone receptors in 60% and positive for HER2 in 39.7%.

### 3.2. Toxicity

The treatment was generally well tolerated and Chemotherapy cycle was completed in all patients except three patients (5%). Nine patients (15%) have undergone any grade 4 of adverse events that two of them went off the study after the second infusion of paclitaxel cycle due to grade 4 paresthesia concomitant with muscular pain and/or diarrhea, which was not tolerable by patients and did not further receive paclitaxel. Besides, two patients experienced paresthesia after last infusion of paclitaxel.

Treatment delayed in 10 patients (16.7%). The cause of delay was nausea, diarrhea, neuropathy, and skin-nail disorders. No decrease in dose was required. Also, one patient withdrew after the tenth infusion of paclitaxel cycle due to hyperosmolar diabetes which was not related to study treatment.

Six patients (10%) were hospitalized due to adverse events such as paresthesia, skin-nail disorders, arthralgia, dehydration, nausea, vomiting, and diarrhea. However, there were no cases of toxicity-related deaths.

Hematological and nonhematological toxicity data are summarized in Table 2.

None of the patients experienced cardiac toxicity or sign of heart failure during treatment and follow-up as initial and posttreatment echocardiography in all patients was normal.

As a consequence of the regular assessment of blood counts, except one patient who experienced grade 3 neutropenia, none of the patients suffered from grade 3/4 hematologic toxicity; however, there was a nearly high rate of grade 1/2 neutropenia (35%), but it was asymptomatic and almost did not modify the treatment plan (there was only one case of grade 3 neutropenia). Likely, it was the reason for the use of G-CSF in all patients. Grade 1/2 anemia was common (75%) as well.

During weekly paclitaxel, sensory neuropathy was a common adverse event 25 (41.7%). Another remarkable nonhematological toxicity grade 3/4 was 14 cases of skin-nail disorder (23.4%). High fraction of patients suffered from muscular toxicity; 12 cases suffered (20%) from myalgia; and 14 cases suffered from (23.3%) arthralgia. Eight patients (13.3%) developed total alopecia.

### 3.3. Follow-Up

At the time of the analysis, the median follow-up period was 27 months. 91.6% of patients were disease-free. Four systemic relapses were observed and three of them are dead.

## 4. Discussion

This trial was designed to assess safety and tolerability of dose-dense epirubicin and cyclophosphamide followed by weekly paclitaxel as adjuvant chemotherapy in node-positive breast cancer patients and it was found that this chemotherapy regimen was well tolerated in terms of low hematologic toxicity which is most likely due to G-CSF support. Also, there were not any cases of cardiac toxicity and nausea and vomiting were controlled easily. The results are consistent with studies which confirmed the feasibility of dose-dense regimens and benefits of weekly paclitaxel [5–9, 11].

Results from the recent studies have demonstrated that accelerated epirubicin or doxorubicin with cyclophosphamide given at 2-week interval with G-CSF support could be well tolerated as same as given schedules over standard 3-week intervals in early breast cancer with fewer grade 3/4 neutropenia [9].

Citron et al. compared standard 3 weekly and accelerated 2 weekly schedules of concurrent doxorubicin and cyclophosphamide followed by paclitaxel or sequential doxorubicin, paclitaxel, and cyclophosphamide. They found that grade 4 neutropenia was more frequent in the standard 3 weekly schedules than the accelerated regimens (33% versus 6%, *P* < 0.0001) [11].

Hamid Reza Mirzaei and colleagues conducted a trial of dose-dense epirubicin and cyclophosphamide followed by docetaxel. 55% of patients suffered from grade 4 toxicity and most common grade 3/4 toxicities included neurosensory, arthralgia, and skin toxicity [14].

Burnell et al. compared three groups of the following:

1. cyclophosphamide, epirubicin, and fluorouracil (CEF),
2. epirubicin and cyclophosphamide every 2 weeks for 6 cycles followed by paclitaxel every 3 weeks for 4 cycles (EC/T),
3. doxorubicin and cyclophosphamide every 3 weeks for 4 cycles followed by paclitaxel every 3 weeks for 4 cycles (AC/T).

There was more nausea and vomiting with the EC/T regimen compared with the other two arms. The rate of febrile neutropenia was the highest in the CEF arm. Cardiac toxicity as reflected by symptomatic congestive heart failure was low but was the highest in the CEF arm [15]. As in our trial, there was more peripheral neuropathy with the taxane-containing regimens.

Mamounas et al. conducted a trial of doxorubicin and cyclophosphamide every 3 weeks for four cycles compared to doxorubicin and cyclophosphamide followed by four additional 21-days cycles of paclitaxel. Most common grade 3 or greater toxicity during paclitaxel therapy included neurosensory toxicity, neuromotor toxicity, arthralgia and/or myalgia, and febrile neutropenia in 15%, 7%, 12%, and 3% of patients, respectively. They confirmed the benefits of incorporating a taxane (paclitaxel) in the adjuvant setting [6].

Neurotoxicity is a major concern in paclitaxel-associated treatment. As expected, we found that a significant fraction of patients had grade 3 or higher of peripheral neuropathy during treatment, but these had resolved in all patients by subsequent follow-up; and nearly a high percentage of patients experienced moderate-to-severe myalgia and arthralgia that were treated symptomatically.

Recently, in a randomized trial by Sparano, four cycles of AC were administered every 3 weeks postoperatively which was followed by one of four taxane-based treatments, specifically “paclitaxel” or “docetaxel” either weekly or every three weeks. They concluded that the group receiving weekly paclitaxel had significantly more moderate-to-severe neuropathy than the group receiving standard therapy which was consistent with our results. Moreover, the group receiving docetaxel every 3 weeks showed significantly more severe neutropenia and its associated complications which were more frequent in docetaxel group than paclitaxel [5].

Ishikawa and colleagues evaluated the feasibility of AC/EC every three weeks followed by weekly paclitaxel or four cycles of three consecutive weekly administration followed by a one-week rest (3 × 4) and reported during AC/EC, (68%) of patients developed grade 3/4 granulocytopenia, compared to (7.8%) receiving 12 PAC and (2.1%) receiving 3 × 4 PAC. Sensory neuropathy was a common adverse event during weekly paclitaxel. Although sever symptoms of grade 3 occurred in only one patient with 12 PAC, grade 1/2 neuropathy occurred in (52.7%) patients receiving 12 PAC and in (54.1%) receiving 3 × 4 PAC [16].

Among the other important findings, a high incidence of neurosensory disorders after repetitive cycles of weekly paclitaxel was also noted; however, it was not so severe for patients to withdraw from the study. This adverse effect was the major problem with long-term treatment with paclitaxel which caused substantial patients discomfort. No differences in nonhematologic toxicities such as neurosensory disorders were observed between 12 PAC and 3 × 4 PAC treatment [16].

Follow-up of patients in this study will demonstrate results for the efficacy of this treatment, which is a secondary endpoint of disease-free and overall survival.

The endpoint was the incidence of grade 4 toxic events. According to the statistical design of the trial, 9 patients (15%) experienced grade 4 toxicity with this chemotherapy regimen.

In conclusion, the present study concluded that dose-dense epirubicin and cyclophosphamide plus weekly paclitaxel with G-CSF support is a feasible and tolerable regimen in node-positive breast cancer patients, particularly with regard to neurotoxicity, the major concern of paclitaxel-associated treatment. However, larger double-blind, randomized controlled trials are needed to generalize this finding.

## Figures and Tables

**Table 1:** Clinical characteristic of patients.

Characteristics	
	Median (range)
Age (years)	49.6 (30–70)
Tumor size (cm)	3 (1–9)
Number of positive nodes	2 (1–37)
Side of involved	No. (%)
Right	28 (46.7%)
Left	32 (53.3%)
Histology	
Ductal	53 (89.8)
Lobular	3 (5)
Others	4 (5.2)
Hormone receptors	
ER	
Positive	43 (75.4)
Negative	14 (24.6)
PR	
Positive	36 (63.2)
Negative	21 (36.8)
HER-2	
Positive	32 (58.2)
Negative	23 (41.8)
Hormone therapy	
Positive	47 (78.3)
Negative	13 (21.7)
Regimen of hormone therapy	
Tamoxifen	28 (59.6)
Others	19 (40.4)

ER: estrogen receptors; PR: progesterone receptors.

**Table 2:** Incidence of toxicities in treated patients.

Toxicities	G2 number (%)	G4 number (%)
Haematological toxicity		
Neutropenia	11 (18.3)	—
Febrile neutropenia	1 (1.7)	—
Anemia	14 (23.3)	—
Thrombocytopenia	3 (5)	—
Nonhaematological toxicity		
Skin-nail disorders	1 (1.7)	3 (5)
Scaling	—	—
Stomatitis	—	—
Hand-foot syndrome	—	—
Erythema	—	—
Paresis	—	—
Paresthesia	2 (3.3)	7 (11.7)
Myalgia	9 (15)	2 (3.3)
Arthralgia	4 (6.7)	2 (3.3)
Nausea	6 (10)	2 (3.3)
Vomiting	—	—
Diarrhea	—	2 (3.3)
Fluid retention	1 (1.7)	—

Any grade 4 event	9 (15)	
